# “Yet Once More”: The Double-Slit Experiment and Quantum Discontinuity

**DOI:** 10.3390/e24101455

**Published:** 2022-10-12

**Authors:** Arkady Plotnitsky

**Affiliations:** Literature, Theory, Cultural Studies Program, Philosophy and Literature Program, Purdue University, West Lafayette, IN 47907, USA; plotnits@purdue.edu

**Keywords:** Bohr discontinuity, Heisenberg discontinuity, Dirac discontinuity, the double-slit experiment, reality, realism, reality without realism

## Abstract

This article reconsiders the double-slit experiment from the nonrealist or, in terms of this article, “reality-without-realism” (RWR) perspective, grounded in the combination of three forms of quantum discontinuity: (1) “Heisenberg discontinuity”, defined by the impossibility of a representation or even conception of how quantum phenomena come about, even though quantum theory (such as quantum mechanics or quantum field theory) predicts the data in question strictly in accord with what is observed in quantum experiments); (2) “Bohr discontinuity”, defined, under the assumption of Heisenberg discontinuity, by the view that quantum phenomena and the data observed therein are described by classical and not quantum theory, even though classical physics cannot predict them; and (3) “Dirac discontinuity” (not considered by Dirac himself, but suggested by his equation), according to which the concept of a quantum object, such as a photon or electron, is an idealization only applicable at the time of observation and not to something that exists independently in nature. Dirac discontinuity is of particular importance for the article’s foundational argument and its analysis of the double-slit experiment.

## 1. Introduction

The role of discontinuity in quantum physics initially emerged as discreteness in the form of the Democritean idea of atomicity (defined by the limited divisibility of matter) with M. Planck’s discovery of the quantum nature of radiation in 1900. Quantum discontinuity, however, gradually revealed itself to be a more complex phenomenon, open to various conceptions and interpretations, just as are, in the first place, quantum phenomena themselves and quantum theory. I suggest in this article that quantum discontinuity may be accounted for by three main types of discontinuity—Heisenberg, Bohr, and Dirac discontinuities—with Heisenberg discontinuity defining nonrealist or, as I shall designate them, “reality without realism” (RWR) interpretations of quantum phenomena and quantum theory, specifically quantum mechanics (QM) and quantum field theory (QFT). (Alternative quantum theories, such as Bohmian mechanics or spontaneous collapse theory, are not considered, except in passing).

These three forms of quantum discontinuity explain and are justified by (along with other key quantum experiments) the double-slit experiment, which I, yet once more, consider in this article. Interminably discussed, it remains the dominant paradigmatic quantum experiment, even though it has acquired some competition during the last half-century, such as those of the Einstein–Podolsky–Rosen (EPR) type concerning quantum correlations. The latter experiments can, however, be shown to be in accord with RWR-type interpretations, specifically the one adopted here, which contains Dirac discontinuity, as considered by this author previously, although Dirac discontinuity, while assumed in these works, was not named as such [1,2,3]. This form of discontinuity is not necessarily found in other RWR-type interpretations, for example, that of N. Bohr, historically the first RWR-type interpretation, although it was intimated by assuming Heisenberg discontinuity by W. Heisenberg in his discovery of QM [4].

Heisenberg discontinuity, defining RWR-type interpretations, places the emergence of quantum phenomena beyond representation or knowledge, in which case I shall speak of the weak form of such interpretations, or even beyond conception, in which case I shall speak of the strong form of such interpretations. This article adopts a strong RWR-type interpretation, assuming, in addition, both Bohr and Dirac discontinuities. The capacity of the mathematics of these theories to predict the outcomes of quantum experiments fully in accord with what is observed, in general, probabilistically (and no other predictions are, in general, possible on experimental grounds), becomes in turn enigmatic—beyond knowledge or even conception of why it does so. We know how this mathematics works (how to use it), but we do not know and perhaps cannot know or even conceive of why it works. Fortunately for us, however, this mathematics does work, regardless of interpretation. 

The possibility of realist interpretations, ideally (as modern physics only deals with such idealizations) representing the properties and behavior of the object considered, in classical physics is due to, or at least emerged from, the circumstance that the concepts of classical physics are mathematical refinements of the concepts of our general phenomenal experience of the world. This refinement, possible and effective in classical physics and (with limitations, as discussed below) in relativity, has proven to be difficult and even impossible to use in quantum theory, even in realist interpretations, which would assume that QM or QFT provides a mathematized representation of quantum objects and processes. Heisenberg discontinuity, defining RWR-type interpretations, precludes any representation or even conception of the ultimate constitution of the physical reality responsible for quantum phenomena. The emphasized phrase indicates a crucial qualification. Classical physics remains essential in quantum theory, including in RWR-type interpretations, as applicable to quantum phenomena, defined by what is observed in measuring instruments and described, along with the observable parts of these instruments, by classical physics. How quantum phenomena come about, and hence the ultimate nature of the reality responsible for them, cannot, in RWR-type interpretations, be described or represented by QM or QFT but only predicted by it, in general, probabilistically. Hence, QM or QFT has, in these interpretations, no physical connections, apart from making these predictions, either to the ultimate nature of the reality responsible quantum phenomena, which defines Heisenberg discontinuity, or, because they are described by classical physics, to these phenomena themselves, which defines Bohr discontinuity.

I call the discontinuity between QM and the emergence of quantum phenomena Heisenberg discontinuity, because it was introduced by Heisenberg along with QM itself, and the discontinuity between QM and the (classical) physics of quantum phenomena “Bohr discontinuity”, because it was introduced as part of Bohr’s interpretation of quantum phenomena and QM under the assumption of Heisenberg discontinuity. Bohr grounded his interpretation (in all of its versions, as he changed his views a few times) in the irreducible role of measuring instruments in the constitution of quantum phenomena and, in the ultimate version of his interpretation, in the strong RWR view, as applied to the ultimate constitution of the reality responsible for quantum phenomena. On the other hand, the behavior of the observable parts of measuring instruments, defining quantum phenomena, was idealized as representable, specifically by means of classical physics. Measuring instruments also have quantum strata through which they interact with quantum objects. Eventually, Bohr adopted the term “phenomenon” to refer strictly to what is observed in measuring instruments as effects of their interaction with quantum objects (e.g., [5] (v. 2, p. 64)).

Thus, combining, as in RWR-type interpretations, Heisenberg and Bohr discontinuities precludes QM or QFT from being connected to and, as it were, makes them discontinuous with either the ultimate constitution of the physical reality responsible for quantum phenomena or that of the quantum phenomena themselves, other than by means of probabilistic predictions concerning the data, classical in character, contained in quantum phenomena. By contrast, in classical physics or relativity, the mathematical formalism of the theory is representationally and hence epistemologically connected to (and connects) and is continuous with both the constitution of the physical reality responsible for the phenomena considered and these phenomena themselves. By the same token, these phenomena can be identified with the physical objects considered, because the interference of measuring instruments can be neglected for all practical purposes. This identification is no longer possible in considering quantum phenomena, in the constitution of which the role of measuring instruments is irreducible.

It is this impossibility that compels the present author to bring Dirac discontinuity into the epistemological architecture of quantum theory, as defined by Heisenberg and Bohr discontinuities, with Bohr discontinuity grounded in the irreducible role of measuring instruments in quantum phenomena, while not assuming Dirac discontinuity. In Bohr’s view, the ultimate constitution of the reality responsible for quantum phenomena (again, physically described by classical physics) was associated with quantum objects, which, on Kantian lines, is different from quantum phenomena because of the irreducible role of measuring instruments in their constitution, the role leading Bohr to Bohr discontinuity, coupled to Heisenberg discontinuity. The interpretation adopted in this article takes a more stratified view of the situation by adding Dirac discontinuity to Heisenberg and Bohr discontinuities and, via them, to RWR-type interpretations, following [1,2,3], although, while adopting the concept of Dirac discontinuity, these works did not use the term as such. This stratification is as follows.

The ultimate RWR reality responsible for quantum phenomena is, by Heisenberg discontinuity, an idealization assumed to exist independently of our interactions with it and, thus, of observation. On the other hand, by Dirac discontinuity, as assumed here, the concept of a quantum object, such as a photon or electron, is an idealization that, while still of the RWR type, only applies at the time of observation, defined as the creation of quantum phenomena by the interactions between the ultimate RWR-type reality and instruments. The latter and quantum phenomena are, by Bohr discontinuity, described by classical physics. Importantly, in all three cases (the ultimate RWR reality responsible for quantum phenomena; quantum objects; and quantum phenomena), one still deals with idealizations associated with and, thus, interpretations of quantum phenomena, supported as these idealizations or interpretations may be by experimental facts, such as that of the impossibility, stressed by Bohr, of observing quantum objects independently of their interaction with measuring instruments. Nobody has ever observed a moving electron or photon as such. It is only possible to observe traces of their interactions with measuring instruments, traces that make it difficult and, in the RWR view, impossible to reconstitute the ultimate nature of the reality responsible for them, whether one sees this reality in terms of quantum objects or only assumes, as, by using Dirac discontinuity, I do in this article, that quantum objects are an idealization applicable only at the time of measurement. Admittedly, the concept of a quantum object can be considered from several alternative, including realist, perspectives (e.g., [6]). Focusing on Dirac discontinuity, I shall put such alternatives aside, apart from Bohr’s (RWR) concept of quantum objects, by way of contrast. While Bohr’s argumentation may be seen as, at certain points, suggesting Dirac discontinuity, Bohr never formulated the corresponding view and appears to have always assumed that the concept of a quantum object is an RWR-type entity applicable independently of measurement.

The reason for using the designation “Dirac discontinuity” is that, while not considered by Dirac himself, it may be seen historically as having emerged from the fact that the existence of the positron and hence antimatter was a consequence of his equation for the relativistic electron. This fact implied that one can no longer maintain the identity of a given quantum object, at least of the type conventionally associated with particle-like ones, such as photons or electrons, within the same experiment. (I qualify because the concept of a quantum field requires further qualifications, discussed below.) This suggests that, given Heisenberg discontinuity as part of an (RWR-type) interpretation, it may be more effective to apply the concept of a quantum object (still as an RWR-type entity) exclusively at the time of observation, technically to something that precedes the observation. As explained below, however, there are more immediate reasons, even in low-energy (QM) quantum regimes, to adopt this view, including in order to more effectively interpret some famous quantum conundrums, such as that of the double-slit experiment. While QFT and, historically, Dirac’s discovery of antimatter bring Dirac discontinuity more sharply into focus, it is the essential part of the RWR-type interpretation of nonrelativistic quantum phenomena and QM adopted here. Do the quantum phenomena of QM or QFT require Dirac discontinuity, or Heisenberg and Bohr discontinuities and RWR interpretations, in the first place? It would be difficult to argue such a case, and it is not my aim to do so. I only claim the consistency of these interpretations and their accord with the experimental evidence currently available.

The next section offers an outline of the RWR view of quantum theory and the three discontinuities defining it. Section 3 considers the double-slit experiment from the perspective thus established.

## 2. Reality without Realism: Quantum Discontinuity and Quantum Epistemology

This section outlines the RWR view of quantum phenomena and QM or QFT, a view that can lead to several interpretations, one of which, defined by adding Dirac discontinuity to Heisenberg and Bohr discontinuities, is adopted here. For simplicity, I shall primarily discuss QM (only referring to QFT when necessary), although my argument applies to and is further supported by QFT.

I begin with a summary of my argument. In RWR-type interpretations, QM is disconnected from both the physical emergence of quantum phenomena, no longer represented by QM, a disconnection defined here as Heisenberg discontinuity, and the observed quantum phenomena, represented by classical physics and not QM, a disconnection defined here as Bohr discontinuity. Quantum phenomena will be assumed to be defined by the fact that, in considering them, the Planck constant, *h*, must be taken into account, putting aside qualifications of this definition, necessary in general but not germane for this article (e.g., [1] (pp. 37–38) and [7]). QM, however, is connected to quantum phenomena and connects them by predicting, probabilistically, the outcomes of quantum experiments. This is fully in accord with what is observed, as no other predictions concerning such outcomes are in general possible, as concerns kinematic or dynamical variables, such as the position or the momentum, or the direction of spin. (Such quantities as mass, charge, or spin are invariant.) These probabilistic predictions are, moreover, only possible by using rules added to the formalism rather than being part of it, such as Born’s rule, which relates (essentially, by using complex conjugation) complex quantities of the QM formalism, defined over ℂ, to real numbers corresponding to the probabilities of quantum events. Representational physical ontologies are still necessary in RWR-type interpretations in dealing with the reality observed in measuring instruments, defining quantum phenomena, which are described by classical physics. On the other hand, the knowledge or even conception of the ultimate nature of the reality responsible for quantum phenomena is no longer assumed to be possible by Heisenberg discontinuity. In addition, in the present view, by Dirac discontinuity, the concept of a quantum object, including that of an elementary particle, such as an electron or a photon, is an idealization (still of the RWR type) only applicable at the time of observation, in contrast to the RWR-type reality ultimately responsible for quantum phenomena, which is assumed to exist independently. Essentially, this means that nature or matter is assumed to exist independently, which amounts to the assumption that it existed before we existed and will continue to exist when we no longer exist. While, however, nature exists apart from us and while quantum phenomena would not be possible without it, they would neither be possible without our interaction with nature by means of our technology and our human ways of observing them. On the other hand, in the present view, nature has no quantum objects. Quantum objects are an idealization created by us in our interactions with nature as manifested in quantum phenomena, and, in the present view, this idealization only applies at the time of observation. Nature only has itself, and when it comes to its ultimate constitution, at least the part of this constitution that is responsible for quantum phenomena, it is beyond our knowledge or even conception in the RWR view.

I shall now outline the concept of reality-without-realism (RWR) and the philosophical view, the RWR view, which grounds the corresponding interpretations, RWR-type interpretations, of quantum theory, such as that of Bohr in its ultimate version. This outline follows [1,2,3], which contain further details. This concept is grounded in more general concepts of reality and existence, which are assumed to be primitive concepts and are not given analytical definitions. By “reality”, I refer to that which is assumed to exist, without making any claims concerning the character of this existence or reality, claims that, as explained below, define realism. The absence of such claims allows one to place this character beyond representation or even conception, whose placement defines the RWR view. I understand existence as a capacity to have effects on the world with which we interact and that we can see, represent, know, conceive of, and so forth. The very assumption that something is real, including that of the RWR type, is made, by inference, on the basis of such effects. 

The RWR view is grounded in the assumption that observable effects of physical reality entail a representation of these effects but not necessarily a representation or even a conception of how these effects come about. Such a representation or conception may not be possible and is not in the RWR view in the case of the ultimate constitution of the physical reality responsible for quantum phenomena. As indicated in the introduction, I shall speak of the weak RWR view of this constitution when it is only beyond representation and the strong RWR view when it is beyond conception. An RWR theory or interpretation, thus, assumes different levels of idealizations of reality, some allowing for a representation or conception and others not. Importantly, in the present view, there is no assumption of a uniform character of the ultimate RWR-type reality considered in either QM or QFT, that is, the assumption of it being uniform and only manifesting itself differently in quantum experiments. This assumption is incompatible with the strong RWR view adopted in this article, which precludes any conception of this reality and, thus, also that of its wholeness or oneness, uniform or not. While each time unknowable or even unthinkable, an RWR-type reality is assumed each time to be different. This is what makes each quantum phenomenon, as an effect of this reality, individual and unrepeatable—unique—manifesting, in turn, the unique nature of this reality each time one encounters it through its effects. One can always repeat the setup of a given experiment, because this setup can be classically controlled, but the outcome of measurement, while ideally the same in classical experiments, will be different in quantum ones, because, as quantum, the interaction between the ultimate constitution of the reality responsible for quantum phenomena cannot be controlled [8] (pp. 697, 700). 

Realist thinking is manifested in the corresponding theories, commonly representational in character. Such theories aim to represent the reality they consider in modern, post-Galilean physics by mathematical models, suitably idealizing this reality. It is possible to aim, including in quantum theory, for a strictly mathematical representation of this reality apart from physical concepts, at least as they are ordinarily understood, say, in classical physics or relativity. It is also possible to assume an independent architecture of the reality considered while admitting that it is not possible either (A) to represent this architecture or (B) even to form a rigorously specified concept of it, either at a given moment in history or even ever. Under (A), a theory that is merely predictive could be accepted for lack of a realist alternative, usually with the hope that a future theory will do better by being a representational theory. What, then, grounds realism most fundamentally is the assumption that the ultimate constitution of reality possesses properties and the relationships between them or, as in (ontic) structural realism, just a structure, the more elemental constituents of which are not defined in terms of properties [9]. Such properties, relationships, or structures may either be ideally represented and hence known or be unrepresentable or unknown, or even unknowable, but still conceivable, usually with a hope that the constitution will be eventually so represented. Most realist theories are representational. In considering physics or science, the concept of realism just outlined is often called “scientific realism.” However, this outline would apply to most forms of realism in science or philosophy that I am familiar with. It does not, of course, cover *all* possible forms of realism: it would be difficult, if possible at all, to do in a single outline. I shall also refer, as is common, to realist theories as ontological. Another common term for realist theories is “ontic”, in part used because “ontology” has other meanings.

Thus, classical mechanics (used in dealing with individual objects and small systems, apart from chaotic ones), classical statistical mechanics (used in dealing, statistically, with large classical systems), chaos theory (used in dealing with classical systems that exhibit a highly nonlinear behavior), or relativity, special and general, are realist theories. While classical statistical mechanics does not represent the overall behavior of the systems considered because their mechanical complexity prevents such a representation, it assumes that the individual constituents of these systems are represented by classical mechanics. In chaos theory, which also deals with systems consisting of large numbers of atoms, one assumes a mathematical representation of the behavior of these systems. Relativity posed major, even insurmountable, difficulties for our general phenomenal intuition, because the relativistic law of the addition of velocities (defined by the Lorentz transformation) in special relativity,$\;s=\frac{v+u}{1+{\left(vu/c\right)}^{2}}$, for collinear motion (*c* is the speed of light in a vacuum) runs contrary to any possible intuitive conception. Our phenomenal intuition cannot conceive of (visualize) this kind of motion. This concept of motion is no longer a mathematical refinement of a daily sense of motion as the concept of motion is in classical physics. Relativity was the first physical theory that defeated our ability to form a phenomenal visualization of an elementary individual physical process. Bohr did not miss this point: “I am glad to have the opportunity of emphasizing the great significance of Einstein’s theory of relativity in recent development of physics with respect to our emancipation from the demands of visualization” [5] (v. 1, pp. 115–11). Emancipation, no less! Special and then general relativity, however, still offer mathematically idealized conceptual representations of the physical reality they considered and, in this respect, allowed this reality still to be available to thought or, as it were, *visible to thought*. Quantum physics, thus, brought this emancipation to a more radical level: that of the *invisible to thought*.

All of the theories just mentioned are based on the assumption that we can observe the phenomena considered without disturbing them and, as a result, identify them with the corresponding physical objects and their independent behavior and (ideally) represent this behavior and (ideally) predict it by using this representation. This is no longer possible when dealing with quantum phenomena, regardless of interpretation, and hence also in the case of realist interpretations of QM or alternative theories, such as Bohmian mechanics, of quantum phenomena. This impossibility grounded Bohr’s interpretation, in all its versions, eventually leading him to his ultimate strong RWR-type interpretation of quantum phenomena and QM, which was grounded in and took advantage of this impossibility. Bohr adjusted, sometimes significantly, his interpretation. This requires one to specify to which version of his interpretation one refers, which I shall do as necessary while focusing on his ultimate RWR-type interpretation in the present interpretation of his interpretation. The designation “the Copenhagen interpretation” requires even more qualifications as concerns whose interpretation it is, say, those of Heisenberg, Dirac, or von Neumann. For this reason, I shall avoid this designation altogether.

As Bohr argued in the Como lecture, which presented his first interpretation of QM, in classical physics and relativity, “our… description of physical phenomena [is] based of the idea that the phenomena concerned may be observed without disturbing them appreciably” ([5] (v. 1, p. 53)). By contrast, “any observation of atomic [quantum] phenomena will involve an interaction [of the object under investigation] with the agency of observation not to be neglected” [5] (v. 1, p. 54)). There is a subtle nature of this contrast: the interaction between the object under investigation and the agency of observation gives rise to a quantum phenomenon rather than disturbs it. Bohr became weary of using the language of “disturbing of phenomena by observation” [5] (v. 2, p. 64). Speaking of “an interaction [of the object under investigation] with the agency of observation”, which cannot be neglected, expresses more precisely the irreducible role of observational technology in the constitution of quantum phenomena. 

Bohr grounded his interpretation (in all its versions) in the irreducible role of measuring instruments in defining quantum phenomena and, in the ultimate version of his interpretation, in the strong RWR concept of reality, as applied to the ultimate constitution of the reality responsible for quantum phenomena, placed beyond conception and made invisible to thought, thus defining Heisenberg discontinuity in its strongest form. On the other hand, the behavior of the observable parts of measuring instruments, defining quantum phenomena (visible to thought and even our immediate sense perception), was idealized as representable, specifically by means of classical physics, thus defining Bohr discontinuity. Measuring instruments, however, also have quantum strata through which they interact with quantum objects. Eventually, Bohr adopted the term “phenomenon” to refer strictly to what is observed in measuring instruments as effects of their interaction with quantum objects [5] (v. 2, p. 64). The ultimate constitution of the reality responsible for quantum phenomena was associated by Bohr, on Kantian lines [10], with quantum objects vs. quantum phenomena observed in measuring instruments.

The interpretation adopted here takes a more stratified view defined by Dirac discontinuity. The ultimate RWR reality responsible for quantum phenomena is an idealization assumed to exist independently of our interactions with it and, thus, of observation. On the other hand, the concept of a quantum object, such as an electron, is an idealization that, while still of the RWR type, only applies at the time of observation, considered the creation of quantum phenomena by the interactions between the ultimate RWR-type reality and instruments. Technically, this idealization applies to something that precedes observation because, by the time of observation as such, the quantum object considered is either destroyed or has left the location of the instrument that enabled the observation [1] (pp. 192–196); [5] (v. 2, p. 57). At least, this is the case if this concept is associated with particle-like observational effects, such as those associated with elementary particles, and measurable quantities, such as mass, charge, or spin, comprising these effects. The concept of a quantum field, if considered as a quantum object, introduces additional complexities, on which I comment below.

As noted from the outset, however, although high-energy quantum regimes and QFT, where the concept of a quantum field, again, becomes essential, offer strong reasons for adopting the concept of Dirac discontinuity and the designation itself [1] (pp. 273–306) and [2], there are more immediate reasons in low-energy (QM) quantum regimes for doing so, including certain key aspects of the double-slit and related experiments, as discussed below. One such reason is suggested, even if not strictly required, by the nature of quantum phenomena as such, regardless of an interpretation, although RWR-type interpretations of this nature make the idea of Dirac discontinuity more compelling. (As stated from the outset, these and most interpretations are those of both quantum phenomena and QM.) In contrast to classical physics or relativity, in quantum physics, in each experimental arrangement, one must, regardless of interpretation, in Bohr’s words, discriminate “between those parts of the physical system considered which are to be treated as measuring instruments and those which constitute the objects under investigation” [8] (p. 701). The difference between them is, however, not uniquely defined. This is expressed as the arbitrariness of the “cut”, as discussed below. Bohr’s main point is clear, however: It is how we set up an experiment that defines what is the object under investigation in this experiment. This object may not be strictly a quantum object: it may be partly classical, which is why Bohr here speaks of “objects under investigation” rather than quantum objects. It can, however, be only partly classical because it must contain, as its part, a quantum object, such as an electron or a photon, or some composite quantum object affecting the observation, to observe quantum effects, the capacity of which defines quantum objects in Bohr’s interpretation (or at the time of measurement in the present view). I discuss this aspect of the situation below. If the object under investigation is a properly quantum object, then what is so observed will of course be defined by the nature of this object, such as an electron or photon, or, again, the stratum of reality so idealized, in terms of the effects it has on measuring instruments, such as those associated with its mass, charge, or spin. The situation still allows for assigning, as Bohr does, quantum objects an independent existence, even if still assuming them to be RWR-type entities, to which no properties can be assigned. On the other hand, the definition of the object under investigation, as always defined by an observation, does suggest that if this object is a properly quantum object and, as such, can never be specified in terms of any properties independently of observation, then the concept of a quantum object may only apply at the time of observation. If the object under investigation is classical, even though our prediction concerning the outcome of an experiment involving it is made by QM, then the object need not be defined by an experiment, because one can assume its properties and behavior to be independent of its interactions with the means of observation. This assumption is, however, never possible for a quantum object in RWR-type interpretations, compelling Bohr to speak of “the essential ambiguity involved in a reference to physical attributes of objects when dealing with phenomena where no sharp distinction can be made between the behavior of the objects themselves and their interaction with the measuring instruments” [5] (v. 2, p. 61).

Two key concepts defining classical physics and relativity, (classical) “causality” and (classical) “measurement”, become no longer applicable in QM in RWR-type interpretations. By “classical causality”, I refer to the claim that the state, X, of a physical system is determined, in accordance with a law, at all future moments of time once its state, A, is determined at a given moment of time, and state A is determined by the same law by any of the system’s previous states. This assumption implies a concept of reality, which defines this law, thus making this concept of causality ontological or realist. There are several reasons for my choice of “classical causality”, rather than just causality, which is used more commonly. The main one is that it is possible to introduce alternative, probabilistic concepts of causality applicable in QM, including in RWR-type interpretations, where classical causality does not apply (e.g., [1] (pp. 207–218)). Some, beginning with P. S. Laplace, have used “determinism” to designate classical causality. I define “determinism” as an epistemological category referring to the possibility of predicting the outcomes of classically causal processes, ideally exactly. In classical mechanics, when dealing with individual or small systems, both concepts become equivalent. On the other hand, classical statistical mechanics or chaos theory are classically causal but not deterministic in view of the complexity of the systems considered, which limits us to probabilistic or statistical predictions concerning their behavior.

In quantum phenomena, deterministic predictions are not possible, even in considering the most elementary quantum systems, such as elementary particles. This is because the repetition of identically prepared quantum experiments in general leads to “different recordings” of the observed data (associated with the kinematic and dynamical variables), and unlike in classical physics, this difference cannot be diminished beyond the limit, defined by *h*, by improving the capacity of our measuring instruments [5] (v. 2, p. 73). “Recordings” refers to both those of the initial measurement, enabling a prediction, and those of the second measurement, which would verify this prediction, a combination that generally defines an experiment in physics. These recordings will be different either if we repeat the whole procedure in the same set of experimental arrangements or if we build a copy of the apparatus and set it up in the same way, as we do to separately verify the outcomes of experiments. This is always possible because the preparations of the instruments could be controlled classically. On the other hand, their interaction with quantum objects (or, in the present view, the ultimate nature of the reality responsible for quantum phenomena and, at the time of measurement, quantum objects) cannot be controlled, which compelled Bohr to speak of “the finite and uncontrollable interaction between the object and the measuring instruments in the field of quantum theory” [8] (p. 700). The respective probabilities of the first and second measurements are independent of each other. The most crucial, however, is the difference in the outcomes of the second (predicted) measurement in repeated setups. One can prepare any given state, say, that of a “spin-up”, as manifested in the corresponding measurement, even though one cannot in general do so in a single experimental preparation, but only by post-selecting the required preparation. By contrast, the outcome of the second (predicted) measurement cannot be controlled at all.

The statistics of multiple repeated experiments performed in both such experimental settings will be the same. On the other hand, an individual quantum experiment cannot be reproduced. By contrast, it is always possible to do so in classical physics, because the interference of measurement can be neglected or fully controlled, at least in principle. All actual data observed in quantum experiments remain classical and can always be communicated, but unlike in classical physics, they cannot be recreated by a different system, which must always combine an apparatus, whose observable parts are described classically, and the ultimate constitution of the reality responsible for quantum phenomena, whose constitution, at the time of observation, allows one to speak of quantum objects. This situation embodies the no-cloning theorem [11,12,13].

It follows that the probabilistic or statistical character of quantum predictions must hold in interpretations of QM or alternative theories of quantum phenomena (such as Bohmian mechanics) that are classically causal. QM or QFT, in RWR-type interpretations, are not classically causal because the ultimate nature of the reality responsible for quantum phenomena is assumed to be beyond a representation or conception. Classical causality would imply at least a partial conception and even a representation of this reality. These circumstances imply a different reason for the recourse to probability in quantum theory in RWR-type interpretations. According to Bohr:

It is most important to realize that the recourse to probability laws under such circumstances is essentially different in aim from the familiar application of statistical considerations as practical means of accounting for the properties of mechanical systems of great structural complexity. In fact, in quantum physics we are presented not with intricacies of this kind, but with the inability of the classical frame of concepts to comprise the peculiar feature of indivisibility, or “individuality”, characterizing the elementary processes [5] (v. 2, p. 34).

The “indivisibility” refers to the indivisibility of phenomena in Bohr’s sense, defined by what is observed in measuring instruments, and the impossibility of considering quantum objects independently, apart from their interactions with these instruments. “Individuality” refers to the assumption, which may be designated as the quantum individuality postulate (correlative to the no-cloning theorem), according to which each phenomenon is individual and unrepeatable, as well as discrete relative to any other phenomenon. 

The concept of quantum measurement adopted in this article is no longer that assumed in classical physics (or relativity) either. It follows, with the additional assumption of Dirac discontinuity, Bohr’s view of measurement, eventually leading him to his concept of a “phenomenon”, referring strictly to what has been observed in measuring instruments. The term “measurement” is a remnant of classical physics or still earlier history, beginning with ancient Greek thinking and the rise of geometry, geo-*metry*, there. In Bohr’s and the present view, a quantum measurement does not measure or, in the first place, does not observe any property of this reality, which it would be assumed to possess before or even during the act of observation. The concept of observation requires a redefinition as well. An act of observation in quantum physics establishes, that is, creates, quantum phenomena from an interaction between the instrument and the quantum object. Then, what is observed as the data or information can be measured classically, just as one measures what is observed in classical physics. In this case, however, what is observed or measured could be associated with the object considered. In the case of quantum phenomena, there is a difference between observations, which construct phenomena, and measurements, which classically measure the physical properties of phenomena. In speaking of “quantum measurement”, I refer to this whole process. 

I shall now explain Bohr’s concept of complementarity, especially, as it appears in his ultimate, strong RWR-type interpretation, where it applies to phenomena in Bohr’s sense of a specified observation registered in measuring instruments. As defined generally, complementarity is characterized by:

(A) The mutual exclusivity of certain phenomena, entities, or conceptions;
(B) The possibility of considering each one of them separately at any given point;
(C) The necessity of considering all of them at different moments of time for a comprehensive account of the totality of phenomena that one must consider in quantum physics.

Bohr never gave the concept a single definition of this type. However, this definition may be surmised from several of Bohr’s statements (e.g., [5] (v. 2, p. 40)). In classical mechanics (in dealing with individual or simple systems), we can comprehend all of the information about each object within a single picture because the interference of measurement can be neglected: this allows us to identify the phenomenon with the object under investigation and establish the quantities defining this information, such as the position and the momentum of the object, in the same experiment. In quantum physics, this interference cannot be neglected and leads to different experimental conditions for each measurement and their complementarity, in correspondence with the uncertainty relations. The situation implies two incompatible pictures of what is observed as phenomena in measuring instruments. Hence, the possible information about a quantum object, the information to be found in measuring instruments, could only be exhausted by the mutually incompatible evidence obtained under different experimental conditions. On the other hand, once made, either measurement, say, that of the position, will provide the complete actual information (manifested in measuring instruments) about the object, as complete as possible, at this moment in time. One could never obtain the complementary information provided by the momentum measurement about this object at this moment in time, because to do so, one would need to simultaneously perform a complementary experiment on it, which is not possible. At the same time, by (B), one can decide to perform either one or the other measurement at any point in time.

Complementarity is, thus, a reflection of the fact that, in a radical departure from classical physics or relativity, the behavior of quantum objects of the same type, say, electrons, or, again, the ultimate nature of the reality responsible for quantum phenomena defined by such objects is not governed by the same physical law, especially a representational physical law, in all possible contexts, specifically in complementary contexts. This leads to incompatible observable physical effects in complementary contexts. On the other hand, the mathematical formalism of QM offers correct probabilistic or statistical predictions of quantum phenomena in all contexts, in RWR-type interpretations, under the assumption that the ultimate nature of the reality responsible for quantum phenomena is beyond representation or even conception. This situation is also responsible for what is known as “contextuality”, which was considered from the RWR perspective in [1,14] and, along different lines, in [15] and in Khrennikov’s extended survey [16]. 

It might be noted, especially in connection with the double-slit experiment, that wave–particle complementarity, with which the concept of complementarity is often associated, had not played a significant, if any, role in Bohr’s thinking, especially after the Como lecture. Bohr was always aware, even in the Como lecture, of the difficulties of applying the concept of physical waves to quantum objects or assuming that both types of behavior, particle-like and wave-like, pertain to the same individual entities, such as each photon or electron itself, considered independently. Bohr’s ultimate solution to the dilemma of whether quantum objects are particles or waves was that they were neither, any more than anything else, in line with Heisenberg discontinuity. Instead, either “picture” refers to one of the two mutually exclusive sets of the discrete individual effects, described classically by Bohr discontinuity, of the interactions between quantum objects and measuring instruments. These (classically observed) effects, which are always discrete or particle-like, may be individual or collective, or wave-like, which are always collective, composed of discrete individual effects. An example of the latter is “interference” effects, composed of a large number of discrete traces of collisions between the quantum objects and the screen in the double-slit experiment in the corresponding setup (when both slits are open and there are no means to know through which slit each object has passed). These two sets of effects may be seen as complementary, including when it comes to calculating the probabilities or statistics for each set of events or, if one takes a Bayesian view, for each event of each set, but in terms of complementary wave and particle behaviors in individual quantum objects. Bohr took advantage of the fact that these two types of effects are always mutually exclusive and require mutually exclusive experimental setups to be observed. Bohr also used the idea of “symbolic waves” as related to the probability or statistics of quantum predictions, in accordance with Born’s interpretation (e.g., [8] (p. 697)). Speaking of waves (which commonly refer to continuous physical processes) in this context requires caution. These “waves” define, in Schrödinger’s term, “expectation-catalogs” concerning possible events that are always discrete [17] (p. 154). What is continuous is the wave function, which does not, however, represent any physical process in this view but is only part of the mathematical machinery through which these expectation-catalogs are established.

One gains further insight into quantum measurements by considering the so-called “cut”, via Bohr’s analysis of it, briefly mentioned above. According to Bohr:

This necessity of discriminating in each experimental arrangement between those parts of the physical system considered which are to be treated as measuring instruments and those which constitute the objects under investigation may indeed be said to form a principal distinction between classical and quantum-mechanical description of physical phenomena. It is true that the place within each measuring procedure where this discrimination is made is in both cases largely a matter of convenience. While, however, in classical physics the distinction between object and measuring agencies does not entail any difference in the character of the description of the phenomena concerned, its fundamental importance in quantum theory… has its root in the indispensable use of classical concepts in the interpretation of all proper measurements, even though the classical theories do not suffice in accounting for the new types of regularities with which we are concerned in atomic physics. In accordance with this situation there can be no question of any unambiguous interpretation of the symbols of quantum mechanics other than that embodied in the well-known rules which allow us to predict the results to be obtained by a given experimental arrangement described in a totally classical way [8] (p. 701).

Before I discuss Bohr’s argument itself, I would like to comment on two common misunderstandings of this and related statements by Bohr. First, Bohr’s statement does not mean that, while observable parts of measuring instruments are described by means of classical physics, the independent behavior of quantum objects is described or represented by means of the quantum-mechanical formalism. This type of view has been adopted by some, for example, Dirac [18] and von Neumann [19], moreover, under the assumption of the classically causal independent behavior of quantum objects, with probability introduced by measurement. This was not, however, Bohr’s view, at least not after he revised his argument in the so-called Como lecture [1] (pp. 67–68); [5] (v. 1, pp. 52–91) and [20] (pp. 122–125). Bohr only says that the observable parts of measuring instruments are described by means of classical physics and that classical theories cannot account for quantum phenomena. However, he does not say that the independent behavior of quantum objects is represented by the formalism of QM. His statement only implies that quantum objects cannot be treated classically, which is a very different claim. The “symbols” of QM only have a probabilistically or statistically predictive role, without, by Heisenberg discontinuity, offering a representation of how quantum phenomena come about. This is very different from Dirac’s and von Neumann’s ontological views. A forceful critique of von Neumann’s unitarity ontology of QM, especially of its non-falsifiable nature, was offered in [21], and the same type of critique would, I would argue, apply to Dirac’s ontological view of QM in his book.

Bohr’s insistence on the indispensability of classical physical concepts in considering measuring instruments is often misunderstood as well. Even though what is observed as phenomena in quantum experiments is beyond the capacity of classical physics to predict, the classical description can and (for us to be able to give communicable accounts of what happens in quantum experiments) must apply to the observable parts of measuring instruments. Bohr instructively commented on this point in his letter to Schrödinger, who expressed doubts concerning Bohr’s insistence on this necessity:

On this point I must confess that I cannot share your doubts. My emphasis [on] the point that the classical description of experiments is unavoidable amounts merely to the seemingly obvious fact that the description of any measuring arrangement must, in an essential manner, involve the arrangement of the instruments in space and their functioning in time, if we shall be able to state anything at all about phenomena. The argument here is of course first and foremost that in order to serve as measuring instruments, they cannot be included in the realm of application proper to quantum mechanics. (Letter to Schrödinger, 25 October 1935, in [22] (p. 511))

The last sense expresses the nature of Bohr discontinuity as equally divorcing the formalism (“symbols”) of QM from any physical description of what is observed in a quantum experiment while, at the same time, enabling the prediction of what is observed. As explained above, however, Bohr also assumed that the instruments also have a quantum stratum through which they interact with quantum objects, whose interaction would indeed not be possible otherwise. This interaction is quantum, and hence, it cannot be observed as such or, in RWR-type interpretations, represented or even conceived of. It is just as invisible to thought as is any other part of the ultimate constitution of the reality responsible for quantum phenomena, which are, by contrast, always visible to thought and even our immediate sense perception.

The situation described in Bohr’s passage under discussion is sometimes referred to as the arbitrariness of the “cut” or, because the concept [Schnitt] was favored by Heisenberg and von Neumann, the “Heisenberg-von-Neumann cut.” As Bohr noted, however, while “it is true that the place within each measuring procedure where this discrimination [between the object and the instrument] is made is… largely a matter of convenience”, it is not entirely true. This is because “in each experimental arrangement and measuring procedure we have only a free choice of this place within a region where the quantum-mechanical description of the process concerned is effectively equivalent with the classical description” [8] (p. 701). Thus, the ultimate constitution of the physical reality responsible for quantum phenomena or quantum objects and in the quantum part of the instruments interacting with quantum objects (whose interaction, again, is responsible for quantum phenomena observed in measuring instruments, the observable part of which is described classically) is never on the measurement side of the cut. All observable properties are, by Bohr discontinuity, those of the observable parts of measuring instruments, described classically but appearing under the impact of quantum objects. 

As noted earlier, it is, in principle, possible to place some part of the classical part of the instrument on either side of the cut as “an object under investigation” concerning which quantum predictions can be made by QM, predictions that are, again, always on the measurement side of the cut. A properly quantum object, again, never is. The “object under investigation” must, however, always contain a quantum part, which allows for a possible interaction with a quantum object, because otherwise, no quantum effect could be observed. Hence, Bohr does not call such a (classical) object a “quantum object”, but only an “object under investigation.” Quantum objects strictly belong to the ultimate constitution of the reality responsible for quantum phenomena. As such, they are, again, always on the other side of the cut. In the present view, by Dirac discontinuity, the concept of a quantum object, while still referring to part of the RWR-type reality, only applies at the time of measurement. It is true that, in certain circumstances, say, when it is far enough from the nucleus (for large quantum numbers), an electron can be treated as behaving classically, in which case, however, it is treated as a classical and not a quantum object, as an approximation that disregards the possible quantum effects of this behavior. The case was discussed by both Heisenberg and Bohr early on. As Bohr noted as early as in the Como lecture (1927):

In the limit of larger quantum numbers where the relative difference between adjacent stational states vanishes asymptotically, mechanical pictures of electronic motion [as orbits] may be rationally utilized [by the correspondence principle]. It must be emphasized, however, that this connection cannot be regarded as a gradual transition toward classical theory in the sense that the quantum postulate [as an essential discontinuity and individuality of quantum phenomena] would lose its significance for high quantum numbers. On the contrary, the conclusions obtained from the correspondence principle with the aid of classical pictures depends just upon the assumptions of the conception of stationary and of individual transition processes are maintained even in this limit. [5] (v. 1, p. 85).

It is important to keep in mind that Bohr now refers to the quantum-mechanical view, which, with Heisenberg, renounces, based on the (at least weak) RWR postulate, the classical, orbital “picture” of stationary still retained in Bohr’s 1913 theory, as well of the transitions, “quantum jumps”, between states, the classical representation of which had already been abandoned by Bohr’s 1913 theory. Bohr’s 1913 theory, thus, abandoned the aim of physically representing such transitions, as neither the time nor direction of each jump could be explained, although it could be predicted probabilistically or statistically. This makes the term “jump” misleading in suggesting some representation of what happens. Electrons do not jump: quantum states (as physical states) discontinuously change, and no representation of how they do this is available. What was responsible for these changes was assumed to be real, but its reality was assumed to be at least beyond representation. It was a reality without realism, in accord, at this stage, at least with the weak RWR view, although intimating the strong RWR view, insofar as no concept of how these transitions occurred appeared to be possible to form either.

With QM, the same situation defined the case of electrons in stationary states. Electrons were not moving in orbits around nuclei: their quantum states (associated with variables other than energy, which remained the same in stationary states) were changing, with these changes ultimately only observable in discrete phenomena. Following Heisenberg, in quantum theory, as QM and then QED and QFT, there were only quantum states, manifested in measuring instruments, and transitions between these states. This was a decisive shift in our understanding of the nature of physical reality. Adopting the view that emerged with QED and then QFT, one might argue that rather than making any transition to a new stationary state, an electron in a given stationary state disappears, and a new electron is born in this new stationary state. Each corresponding measurement will detect a different electron, in accord with Dirac discontinuity. The wave function of QM formalism for an electron in an atom can be recast, quite elegantly, in terms of annihilation and creation operators, as are used in QFT. In strong RWR-type interpretations, there exist strata of reality ultimately responsible for quantum phenomena, but these strata cannot be attributed any specifiable states between experiments, in which these states still only manifest themselves in their effects observed in quantum phenomena, but not as such.

Dirac discontinuity, thus, gives a new dimension to the quantum-mechanical situation (in all energy regimes) described by Bohr via the shifting cut between the object under investigation and the measuring instruments, and thus the application of the QC principle. If a quantum object is only an idealization defined by measurement or, again, more accurately, an observation, rather than something that is assumed (even as an idealization) to exist independently, could one still speak of the same quantum object, say, the same electron, in two or more successive observations? Consider two position measurements in the double-slit setup, the first defined by a slit in a diaphragm through which an electron, emitted from some source, may be assumed to be registered to pass by some counter, and the second defined by a collision between it and a silver bromide screen at some distance from the diaphragm. Each of these two measurements defines an electron with the same mass, charge, and spin in two different positions at two different moments in time. The case can be given a strictly RWR interpretation, insofar as all of these properties are, physically, those of measuring devices, assumed to be impacted by quantum objects, rather than those of these objects themselves, placed beyond representation or conception. The question is: Do these two measurements (or that corresponding to an initial emission of an electron in the double-slit experiment) register the same electron? Rigorously speaking, if the concept of a quantum object is only applicable at the time of observation, then a prediction based on a given measurement and the new measurement based on this prediction could only concern a new quantum object and not an object that we measured earlier in making a prediction. Accordingly, one deals with two different quantum objects: two different electrons, for example. To consider them as the same electron is, however, a permissible idealization in low-energy (QM), or low-energy QFT, regimes (still statistical in nature, because no detection of anything can occur in the second measurement). On the other hand, as noted, speaking of the same electron in successive measurements in high-energy (QFT) regimes is meaningless, because these measurements can register quantum objects of different types, such as an electron in the first and a positron or photon in the second [1] (pp. 279–292); [2]. QFT, thus, supports adding Dirac discontinuity to Heisenberg and Bohr discontinuities in RWR-type interpretations. As I argue, however, there are reasons to do so in low-energy (QM) regimes, reasons emerging as early as Bohr’s 1913 theory, but especially in view of the complexities found in the double-slit and other paradigmatic quantum experiments. 

## 3. The Double-Slit Experiment: Quantum Discontinuity and Quantum Probability

This section offers an understanding of the double-slit experiment in the strong RWR-type interpretation of quantum phenomena and QM adopted here, as based on Heisenberg, Bohr, and Dirac discontinuities. Reciprocally, the double-slit experiment reveals the features of quantum phenomena to which this interpretation responds.

The experiment does not depend on QM, which, however, correctly predicts the phenomena observed therein, keeping in mind that photons are relativistic objects and require QED. Rather than only with radiation, with which it was initially concerned, the double-slit experiment can be, in principle, performed with all quantum objects. The experiment was first performed as a quantum experiment with anything other than light only in the 1960s. It has functioned previously as a thought experiment, without much doubt that it could, in principle, be performed on any type of quantum object, following L. de Broglie’s 1923 conjecture that the wave–particle duality (not the same as complementarity!) found in the case of radiation would apply to other elementary constituents of nature, such as electrons. The conjecture was soon experimentally confirmed by the discovery of electrons’ diffraction in crystals. This confidence was further supported by other key quantum experiments that were performed and that exhibited the key features of quantum phenomena exhibited in the double-slit experiment. As a classical experiment, Thomas Young’s double-slit experiment with light has been around since 1801, when it was performed to resolve the dispute as to whether light was composed of particles (according to Newton’s corpuscular theory) or was formed by waves traveling through some form of ether. The interference patterns found in the experiment appeared to have answered the question in favor of the wave theory, which became prevalent before Planck’s discovery of his black-body radiation law, and related developments of quantum theory brought this question back to the center stage of fundamental physics. 

The double-slit experiment was first performed with electrons in the 1960s by Claus Jönsson and with “one electron at a time” by Pier Giorgio Merli in 1974. In 2002, Jönsson’s experiment was voted “the most beautiful experiment” ever performed by a poll conducted in Physics World (September 1, 2002), just edging past Galileo’s experiment with falling bodies. Young’s original experiment made the top 10 as well: it came in 5th, following Newton’s decomposition of sunlight with a prism. One may not claim as much for the double-slit experiment, whether concerning its beauty (a more subjective matter, as Physics World acknowledged) or even its archetypal significance in quantum physics. It has formidable rivals that are nearly as famous and have been used equally well for illustrating the famously strange features of quantum phenomena. Among them are the Stern–Gerlach experiment, various experiments in quantum interferometry (e.g., using the Mach–Zender interferometer), experiments with half-silvered mirrors, or the EPR-type experiments. The latter, especially in Bohm’s version (dealing with discrete variables), has been used in quantum-foundational discussions as often as, if not more often than, the double-slit experiments during the last half-century, following Bell’s theorem. 

Still, the double-slit experiment remains the most famous quantum experiment and is most frequently used to illustrate the conundrums of quantum physics. An additional advantage (for a more general audience) is that it can be more easily explained qualitatively without any technical knowledge of QM. Properly predicting the quantitative data associated with the outcomes of physically performed (or simulated) experiments would of course require the mathematical formalism of some quantum theory, such as QM. The experiment also manifests the uncertainty relations and the complementarity of the position and momentum measurements in the two corresponding setups of the experiments, with the first case allowing one to know, at least in principle, through which each object passes and with the second case precluding such knowledge (speaking in classical terms). Finally, correlatively to the uncertainty relations, the experiment exhibits the unavoidable probabilistic and statistical aspects of predictions concerning quantum phenomena, including the relationships between randomness and probability, and between randomness and a correlational order, an order, admittedly, more pronounced in EPR-type experiments.

The double-slit experiment was central to Bohr’s thinking and to his exchanges with Einstein, including those concerning the EPR experiment [8,23]. It did not figure in Bohr’s Como lecture of 1927, which introduced complementarity, or in the preceding work on quantum mechanics by Heisenberg, Schrödinger, and others. The experiment, however, entered Bohr’s exchanges with Einstein immediately thereafter (e.g., [5] (v. 2, pp. 41–42)). For Bohr, it was one of the primary means for checking our claims concerning quantum phenomena and QM, which properly predicts the numerical data found in the double-slit experiment and thus responds to the character of the phenomena observed therein. While, as noted above, the double-slit experiment was not actually performed until later, other quantum experiments that had been performed in the meantime could be considered as equivalent to it with respect to the key features of the quantum phenomena at stake. This fact enables one to use the double-slit experiment as a thought experiment in theoretical arguments ([8] (p. 698), note). This is not unusual in dealing with thought experiments. The EPR experiment, as originally proposed by EPR, cannot be performed in a laboratory, which, however, has never put its legitimacy in question for the theoretical arguments concerning or based on it. Related experiments, famously those by A. Aspect based on Bohm’s version of the EPR experiment for spin [24], were subsequently performed, as were experiments statistically approximating the EPR experiment. It may be opportune to note that Aspect just shared a Nobel Prize with J. Clauser and A. Zeilinger, who performed key related experiments. Bohr often used the fact that any attempt to circumvent Heisenberg’s uncertainty relation, Δ*q*Δ*p* ≅ *h*, leads to this type of inconsistency with the double-slit experiment. Bohr saw the uncertainty relations as experimentally given, a law of nature, correlative to (which is, of course, not the same as) complementarity, and the fact that they can be derived from QM as only further testimony that QM adequately reflects the data observed in quantum experiments. The uncertainty relations and the data observed in the double-slit experiment are equivalent or at least correlative to each other.

This circumstance was used by Bohr throughout his arguments, especially in his exchanges with Einstein (e.g., [5] (v. 2, pp. 43–47, 52–61); [8]). In the EPR paper, co-authored with B. Podolsky and N. Rosen [23], and other communications, Einstein argued that QM, regardless of interpretation, is incomplete because the EPR experiment (so he contended) allowed one to claim that the exact value of both conjugate variables, the position, *q*, and the momentum, *p*, could be assigned to a quantum object, while QM allowed for no such assignment, in view of the uncertainty relations. Alternatively, if complete, he argued, QM would be nonlocal in the sense of allowing an instantaneous physical action at a distance and, as such, in violation of relativity. Bohr, by using the double-slit experiment as one of his main tools, counterargued that EPR’s argument was based on assumptions that were inconsistent (or at least ambiguous) in view of what could in fact be established on the basis of quantum phenomena, including those considered in the EPR experiment. This allowed him to see QM as both complete within its proper scope (as complete as possible given what is experimentally observed) and local in this sense, or at least that EPR did not demonstrate otherwise [8]. While the debate concerning the subject and the EPR–Bohr exchange, including Bohr’s use of the double-slit experiment, has continued and is still ongoing, I shall put it aside, because it does not affect my argument concerning quantum phenomena and QM in RWR-type interpretations, interpretations that would have never satisfied Einstein. Einstein acknowledged that the EPR argument would not apply if QM were assumed to be only a statistical theory of ensembles, rather than a realist and deterministic theory of individual quantum systems, neither of which would be acceptable to him in a fundamental physical theory (e.g., [25] (pp. 105, 155)). He still believed, however, on the basis of EPR-type considerations, that, if local, QM is incomplete as a theory of individual quantum systems because it does not predict everything that could be established as real (e.g., [1], (pp. 227–272)).

Considering, for simplicity, the case of photons, the experimental arrangement defining the double-slit experiment consists of:

(1) A source, such as that of a monochromatic light, which makes it possible to emit photons one by one;
(2) At some distance from the source, a diaphragm with two slits (B and C), widely separated;
(3) Finally, at a sufficient distance from the diaphragm, a screen—a silver bromide photographic plate—where the traces of these collisions become recorded.

**Figure 1:**
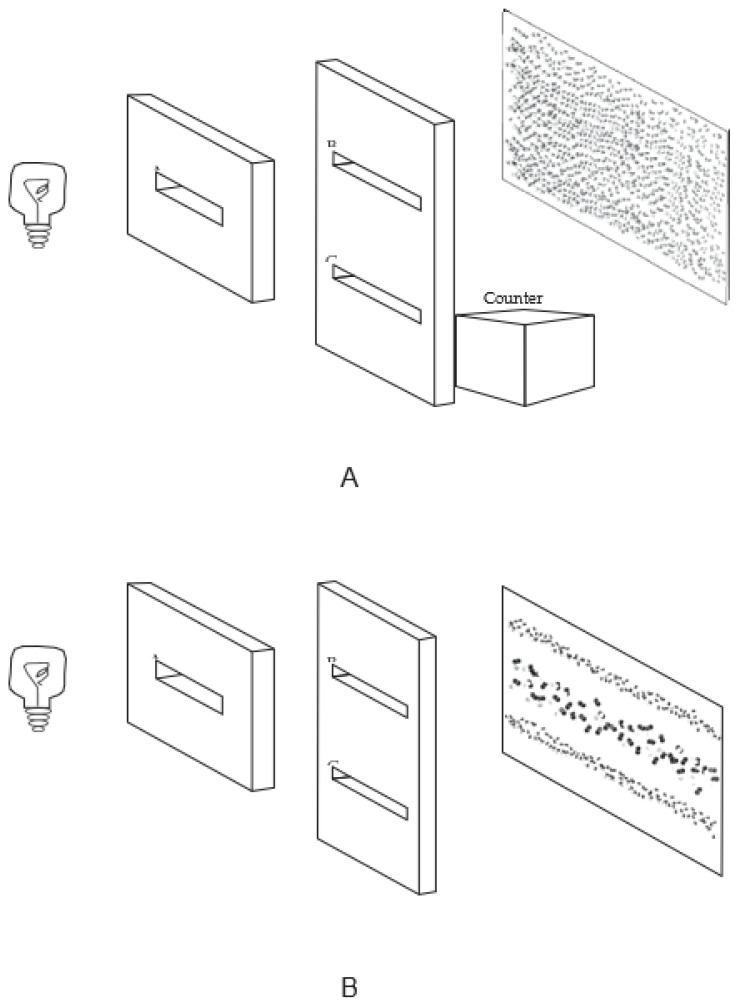
Two setups (**A**,**B**) of the double-slit experiment, symbolically representing, following Bohr [5] (v. 2, pp. 48–49), that one can only observe, *see*, the (“heavy”, humanly made) measuring devices and the traces left by the interactions between them and quantum objects, but not what happens in between, which is beyond conception and thus is *invisible to thought*.

Bohr sometimes used an additional diaphragm with a single slit to define the initial stage (the preparation) of the experiment, as I do here (Figure 1). As noted, one can only observe such traces as effects of processes involving certain types of physical objects or reality, the existence of which we infer from these traces. In each event, we can only observe a mark, which we infer to be a trace left by a “collision” between a quantum object and the screen. Each such trace suggests an image of a collision between a very small object, idealized as a particle in classical physics, and the screen, hence the idea that the individual quantum phenomena involved may be associated with the particle-like behavior of quantum objects. This need not and, in the present view, does not mean that quantum objects are particles any more than they are waves in the sense of classical physics. What is observed as a mark or a trace is a product, or effect, of processes involving millions of atoms. 

Two setups of the double-slit experiment are considered, in each of which a sufficient number (about 70,000 are required to observe interference effects) of photons emitted from a source are allowed to pass through the slits and collide with the screen. In the first setup, A (Figure 1), we can, at least in principle, know through which slit each particle passes; in the second setup, with both slits open, we do not and, more significantly, in principle, cannot know this. This is an important qualification, which plays a key role in several more subtle versions of the double-slit experiment, such as the “quantum eraser” experiment [26]. (The type of “knowledge” may also be considered as the ability to predict which slit could, in principle, be passed through (assuming no faster-than-light influences) based on particle state preparation alone [27].) We can have this knowledge by installing devices, such as counters, which allow us to do so without appreciably disturbing the course of each individual run of the experiment (defined by an individual emission from the source or passing it through the slit in the first, single-slit diaphragm, installed before the one with two slits) in this setup. Such devices are sometimes called the “which-path” or “which-way” devices, terms based on a classical concept, ultimately inapplicable to “what actually happens”, whose expression, as discussed earlier, is not applicable either, at least in RWR-type interpretations. One can also close one of the slits for each such run, which allows each object only to go through one slit. A given quantum object could be blocked by the diaphragm, but these runs of the experiment are discounted. There are experiments, as discussed below, that allow us to “channel” each particle in a more controlled way in each individual run of the experiment, keeping in mind that, while the term (noun) channel applies to the experimental arrangement, one cannot, in the RWR view, speak of a particle passing through a channel. In this setup, quantum objects behave in a particle-like manner both individually and collectively: that is, as just explained, the observed random pattern of collisions is similar to that which would appear if we conducted an analogous experiment with classical objects, idealized as particles. The interference pattern, which occurs in the second setup, never appears. As noted, merely setting up the apparatus in a way that such knowledge could, in principle, be possible, even if not actually obtained, would suffice.

In the second setup, B (Figure 1), the traces of collisions between quantum objects and the screen will, once a sufficient number of photons hit the screen, form an “interference” pattern—a pattern similar (but, by virtue of its discrete individual constituents, not identical) to that produced by the traces of wave processes in an appropriate medium. The interference pattern will appear, in principle (there could be practical limitations), regardless of the distance between slits or the time interval between the emissions. This interval can be made sufficiently long for each emission to take place after the previously emitted object has reached the screen and is destroyed by its collision with the latter, which makes the appearance of the interference pattern especially remarkable.

As an actual physical phenomenon or a set of actual individual phenomena (each discrete relative to each produced by many repeated experiments), this interference pattern may be associated with the idea of waves, but in the RWR view, only metaphorically. Such wave-like effects are more pronounced and more suggestive of physical-wave propagation when we deal with very strong beams consisting of very large numbers of photons following one another in quick succession, the effects of which were initially responsible for wave theories of light, culminating in Maxwell’s electrodynamics. This continuity, however, still ultimately comprises a (very large) set of discrete phenomena.

Indeed, the language of interference may not be suitable here. Interference between what and what? Dirac, in this connection, argued that “each photon… interferes only with itself. Interference between different photons never occurs” [18] (p. 9). Dirac did not clarify how and in what sense each photon does so, as against why there can be no interference between different photons because energy conservation would be violated, which he does explain. As is seen below, however, one can give the first statement a rigorous meaning, including along RWR-type lines. The “correlational pattern”, that is, something that refers to a correlated, ordered (rather than random) distribution of traces, would be a better term, and it can be further justified by other correlational data characterizing quantum phenomena, such as those in the experiments of the EPR type. The interference pattern is, however, an established term that emerged for obvious historical reasons and is unlikely to be changed, in part because the belief in one or another form of physical waves in quantum physics continues to persist. In each individual run of the experiment, the actually observed phenomenon (a mark on the screen) is always particle-like; the interference pattern only emerges in the first and only the first setup just considered out of multiple individual events (one needs about 70,000), as observed in the famous experiments, showing the gradual emergence of this pattern, by A. Tonomuro and his team [28]. This well-recognized fact does not prevent arguments to the effect that the unobserved, or even unobservable, behavior of individual quantum objects can be wave-like, specifically in the situation when the interference pattern or analogous phenomena appear. I consider individual events associated with the slits and individual quantum objects below. I shall note now, however, that there is no experiment that allows one to ever observe individual quantum objects as passing through both slits, in whatever manner, even though it is also difficult and, arguably, in turn, impossible to say (in classical language) that a quantum object can only, in all circumstances, pass through one slit or one channel in other quantum experiments (such as beam-splitter experiments). 

The situation is equivalent to the uncertainty relations and the complementarity of position and momentum measurements, as nicely explained by R. Feynman [29] (v. 3, pp. 1–11). The emergence of the interference pattern may be properly correlated with the possibility of the (ideally) precise momentum measurements for each quantum object involved in the corresponding setup, while the lack of the interference pattern is properly correlated with the possibility of the (ideally) precise position measurement for each quantum object in the alternative setup. Both outcomes, however, and the possibility of measuring both quantities are mutually exclusive or complementary. As noted, this argumentation was used by Bohr throughout his exchanges with Einstein to counterargue Einstein’s criticism of QM (e.g., [5] (v. 2, pp. 43–47, 52–61); [8]). By the same token, the situation is equivalent to the probabilistic and (statistically) correlational nature of our quantum predictions, with correlations manifest in the interference-pattern setup of the double-slit experiment, whose pattern, however, and hence the corresponding correlational order are, again, formed by the accumulation of *random* individual events [1] (pp. 253–256). The provenance of any single event can never be certain, and no single run of the experiment is repeatable. A single event registered by a counter cannot be used to establish unconditionally that an object passed through a slit any more than can any given trace on the screen. These circumstances reflect one of the greatest mysteries of quantum phenomena: How can random individual events, under certain circumstances, give rise to an order, even if only a (statistical) correlational order? [1] (pp. 253–256).

The situation manifested in the double-slit experiment (or equivalently in other paradigmatic quantum experiments) is often referred to as the quantum measurement paradox. As I argue here, however, it is only paradoxical if one tries to understand it in classical terms or ways of thinking, including, and in the first place, as concerns “measurement”, assumed to measure some pre-existing property of a quantum object rather than as a creation of a new phenomenon, each time unique, observed in a measuring instrument, giving us data that can be (classically) measured. The behavior leading to the effects observed in the double-slit experiment cannot be exhibited by the same classical entities, even in different circumstances, nor, of course, can one phenomenally conceive of entities that would be simultaneously particles and waves, or continuous and discontinuous, to begin with. (Even in Bohmian theories, where both concepts are used in describing the behavior of quantum objects, these concepts are not fused in a single entity: a wave accompanies or/and guides the particle in question, following de Broglie’s idea.) Classical objects exhibiting different observed behaviors leading to the two incompatible kinds of observable effects are described by two rigorously different types of theories—by classical mechanics in the case of particle-like objects and by classical electrodynamics in the case of radiation. This is why Planck’s discovery that radiation could, under certain conditions, behave in a particle-like manner was such a shock. One might speak, as some have, of a quantum conundrum, using the term to refer to something difficult to explain. For Bohr, or following him here, the double-slit and related experiments reflect the essential nature of quantum phenomena and the situation they define in fundamental physics. As experimentally observed and mathematically predicted by QM, this situation needs to be logically explained by methods other than (epistemologically) classical means, used in classical physics or relativity. This necessity led Bohr to his strong RWR-type interpretation, which combines Heisenberg and Bohr discontinuities. In the present view, if not that of Bohr, Dirac discontinuity adds a new dimension and indeed form to this alternative logic, as I shall now explain.

I would like to consider, first, following Leggett’s lucid account, a “schematic process”, which can be physically realized as an experiment in quantum interferometry, epistemologically essentially equivalent to the double-slit experiment [30]. In this experiment, we consider the initial state *A*, two possible intermediate states *B* and *C*, and then final states *D* or *E*, following *B*, and *E* or *F*, following *F*, where *B* and *C* are analogous to the slit in the double-slit experiment. Schematically:

         I*_D_*

  I*_B_*

       I*_A_*          I*_E_*

  I*_C_*

         I*_F_*

In the present view, just as in the case of the double-slit experiment, these states are only registered as events in measuring instruments, designated in by I*_X_*, in the absence of any knowledge or even conception of what happens between these events, in contrast to Leggett’s own diagram, which does not include instruments, although he assumed their role [30] (p. 940). (The diagram is symbolic and does not aim to imply that instruments are arranged in space in any particular way, but only that they register these events.) For the moment, however, I shall, following Leggett, provisionally speak in classical-like terms while keeping in mind this and other RWR-type qualifications, ultimately leading to the impossibility of doing so. (Leggett uses the term “microsystem”, in part because of his argument for “macrorealism”, which is, however, a separate subject.) Each quantum object involved in this experiment goes through one of the two possible channels, or possibly both, which is equivalent to assuming that a quantum object goes through both slits in the double-slit experiment. Although the second is a more complex assumption to make, neither assumption applies in Dirac discontinuity. Leggett’s setup allows one to take into account each individual run of the experiment, because we do not need to discount those objects that collide with the diaphragm in the double-slit experiment and do not pass the slit. According to Leggett:

First, we arrange to block the path via state *C*, by some appropriate, but leave the path via state *B* open (in particular, we do not attempt to insert any kind of measurement apparatus to check directly whether the system has passed via state *B*); and to detect the system arriving in state *E*. When we carry out a large number of trials (say 10^6^) under these conditions, in each of which a single microsystem starts in state *A*, and record the number of cases in which this miscrosystem is found to have reach the state *E*. Let this number of [sic!: be] $N_{E}^{\left(B\right)}$. We then repeat the experiment, but this time blocking state *B* and leaving the path via *C* open; let the number arriving in state *E* under these conditions be $N_{E}^{\left(C\right)}$. Finally, we repeat the experiment (again with 10^6^ runs) but now with neither of the intermediate stated blocked; let the number recorded as reaching *E* under this conditions be $N_{E}^{\left(B\right)}$. The striking feature of the experimentally observed result is, of course, summarized in the statement

$$N_{E}^{\left(B+C\right)}\;\neq \;N_{E}^{\left(B\right)}+N_{E}^{\left(C\right)}$$

In words, the number reaching *E* via “either *B* or C” appears to be unequal to the sum of the number reaching *E* “via *B**”*** or “via *C*.” As we know, the outcome may quantitatively explained if we associate with each of these various processes an [probability] amplitude *A*, whose square [by Born’s rule] gives the probability of the process and assumed that it is [these are?] amplitudes that are additive [and not probabilities]. [30] (p. 940).

In the present view, it would be more accurate to say that, rather than “quantitatively explained”, this outcome is reflected in the formalism and Born’s rule in this way. The situation is equivalent to the emergence of the interference pattern when both slits are open in the double-slit experiment. In the absence of any means of establishing through which slit each particle passes or, again, could in principle have passed, or in any situation in which the interference pattern is found, one cannot assume that an object has necessarily passed through either B or C on its way to the screen. If we do, the above probability sum law would not be obeyed, and the conflict with the interference pattern will inevitably emerge, as Bohr noted (e.g., [5] (v. 2, pp. 46–47); [8]). One can put it as follows. In calculating the probabilities of the outcomes of such experiments, we must take into account the possibility of an object passing through both states B and C (or through both slits in the double-slit experiment) when both are open to it. As stated above, however, there have been no experiments performed so far that would allow us to verify this assumption, while any attempt to establish through which slit a given object passes will inevitably disallow the emergence of an interference pattern. At the same time, it is equally difficult and, arguably, in turn, impossible to argue that the object goes through only one slit with both open, especially in the experiments of the type considered by Leggett or similar experiments, such as those using Mach–Zender interferometers.

If, however, one assumes Dirac discontinuity, neither of these two assumptions is possible because one can only speak of a quantum object of any kind (even if it is assumed to be beyond conception, as in the present view) at the time of observation, defined as the creation of a phenomenon observed in measuring instruments, and not otherwise. One, again, never observes the actual “passing” of the object either through one or through both channels in any quantum experiment: one can only register events, amplified to the level of observed phenomena, that happen after an object is beyond either or both channels. Hence, in the present view, there is nothing that can be said about what happens between this event and an earlier observable event, which always happens before the regions of these channels. Leggett concludes his analysis as follows:

In the light of this result, it is difficult to avoid the conclusion that each microsystem in some sense samples both intermediate states B and C. (The only obvious alternative would be to postulate that the ensemble as a whole possesses properties in this respect that are not possessed by its individual members—a postulate which would seem to require a radical revision of assumptions we are accustomed to regard as basic.) This is what I mean by saying that in quantum mechanics all possibilities are left open.

On the other hand, it is perfectly possible to set up a “measurement apparatus” to detect which of the intermediate states (B or C) any particular microsystem passed through. If we do so, then as we know we will always find a definite result, i.e., each particular microsystem is found to have passed either B or C [reaching D or F, respectively]; we never find both possibilities simultaneously represented. (Needless to say, under these [different] physical conditions we no longer see any interference between the two processes.) This is what I mean by saying that in macroscopic experience a definitive outcome is always selected. (Clearly, we can read off the result of the measurement only when it has been amplified to a macroscopic level, e.g., in the form of a pointer position.) [30] (pp. 940–941)

This “behavior” may well be as remarkable as Leggett finds it to be, with either of the two invoked alternatives difficult to entertain. Other standard locutions include strange, puzzling, mysterious, and incomprehensible, for reasons that Leggett’s statement makes apparent. All of these reasons are, however, defined by classical ways of thinking. How do quantum objects “know”, individually or (which may indeed be even more disconcerting, as Leggett notes) collectively, without “knowing” this individually, that both channels or slits are open and no counters are installed or, conversely, that counters are installed to check through which slits particles pass and modify their behavior accordingly? Attempts to conceive of this behavior in terms of the physical attributes of the quantum objects themselves, or even in terms of the independent existence of individual quantum objects (as against only in terms of some unspecifiable or even inconceivable ultimate reality responsible for quantum phenomena), appear to lead to unacceptable or at least highly problematic consequences. Among such consequences are logical contradictions; incompatibility with one aspect of experimental evidence or the other; a strange or mysterious behavior of quantum objects; difficult assumptions, such as attributing volition or personification to nature in allowing particles individual or collective “choices”, such as the one proposed by Leggett; or the nonlocality of the situation, “a spooky action at a distance” incompatible with relativity. Yet another possibility to explain the situation in a classical-like way would be a retroaction in time, which is entertained by some. Leggett offered his own further analysis of the paradoxical, or not, nature of this situation as part of his argument for “macrorealism” (which would, essentially, imply that QM only accounts for the microscopic and not macroscopic reality). I shall put this analysis and Leggett’s argument for macrorealism aside, because he only considers ontological views at either level (in QM, roughly, following von Neumann’s unitary ontology, mentioned above [19]) and does not address nonrealist alternatives, certainly not anything along RWR lines. He is, I might note, also in error in fusing Heisenberg’s and Bohr’s views via the idea of “potentially” [30] (pp. 942, 951). The idea was used by Heisenberg in his later writings (e.g., [15]; [31] (p. 44)), although, importantly, not at the time of his discovery of QM. It was, however, never adopted by Bohr and may even be argued to be incompatible with Bohr’s interpretation in any of its versions, but especially his ultimate, strong RWR-type one.

For the moment, RWR-type interpretations, especially strong ones, beginning with that of Bohr, do not make any of the classical assumptions listed above. They also expressly exclude a retroaction in time or backward-in-time causality in our interaction with the world in the case of quantum phenomena or in general [1] (pp. 207–218). Nor by Heisenberg discontinuity do they make any ontological assumptions, physical or mathematical (for example, in terms of the formalism of QM or QFT), concerning the independent nature and behavior of quantum objects or, in the present view, which assumes Dirac discontinuity, the ultimate constitution of the physical reality responsible for quantum phenomena. They only use and, by Bohr discontinuity, require classical assumptions in considering quantum phenomena. RWR-type interpretations, and they are, again, interpretations, allow one to avoid these paradoxes more easily, I argue, if Dirac discontinuity is added to the RWR-type architecture of physical reality in quantum theory.

The main reason for adopting Dirac discontinuity is the question of what happens, and within what limits and in relation to what one can speak of something that “happens”, in an individual run of the double-slit or related experiments when both channels are open. To reprise the key aspect of the situation, on the one hand, there has thus been no observation that would register, say, again, a photon ever passing through both channels in the manner of a wave. On the other hand, the data observed in individual quantum experiments, especially in the beam-splitter-type ones, make it very difficult and even impossible to assume that a photon always “goes” through either one channel or another (however such channels are established), just without us being able to know through which it passes when both channels are open. QM or QED, again, only predicts, correctly, the statistics of the outcomes. While, as noted, one can speak of these channels as open because they are part of the experimental arrangement, classically describable, speaking of photons as passing through these channels (either one of them or both) is impossible in RWR-type interpretations, because we cannot speak of what happens between observations or even use the word “happens.” It is only possible to assume that we can register the position of the photon next to the single slit in the first diagram as the initial stage of the experiment and the final event in which the photon hits the screen, but there is nothing that can be said or known or even conceived of what happens in between. If both slits are open and there is no means to register events associated with a single slit, neither the statement that the photon passed through one (or the other) slit nor that it passed through both is any more applicable than any other statement. We cannot speak of what happens between the initial and the next observation, nor, in the present view, represent it otherwise, including mathematically. Bohr represented this situation in his famous drawings, in “Discussion with Einstein on epistemological problems in atomic physics” (1949), by depicting heavy measuring instruments with traces of their interactions with quantum objects, without any depiction of what happens between experiments, in accord with the RWR view, which had been adopted by then (e.g., [5] (pp. 48–49)). My diagrams here follow this strategy.

A photon seems to interfere with itself and only with itself and never with other photons, as Dirac said. Is it a wave, then? This is not necessarily so and is expressly not the case in the present strong RWR-type interpretation, accompanied by Dirac discontinuity (which is not related to this statement by Dirac). Given Dirac discontinuity, the concept of a photon, or a quantum object in general, is only applicable (even if, as in the present view, still something beyond conception) at the time of observation, which always occurs after any “passing” of the channels is left behind. As noted, technically, the interaction between a quantum object and the instrument precedes an observation, but it would still occur after the “passing” of the channels. Once again, however, any observation always registers only one photon, a trace of one photon, at one point in space and at any given point in time. One never observes a single photon splitting into two in beam-splitter experiments either (there are no traces permitting such claims): the beam splitters split beams and not individual photons. Hence, when both channels are open, one cannot speak either of a photon going through only one channel, in the manner of a particle, or of going through both, in the manner of a wave. One cannot make either assumption. One can, however, make an assumption that one cannot make assumptions concerning what happened between observations. In fact, as emphasized throughout this article, even the concept of an object passing through a single open channel (with the other one closed) is inapplicable in the RWR view, except that in this case, one cannot consider that the object can pass through both channels, as there is only one open channel. Channels themselves are seen as part of the experimental arrangement and thus described in language. In Heisenberg’s words: “There is no description of what happens to the [quantum] system between the initial observation and the next measurement… We wish to speak in some way about the structure of the atoms and not only about ‘facts’—the latter being, for instance, the black spots on a photographic plate or the water droplets in a cloud chamber. However, we cannot speak about the [constitution of] atoms in ordinary language” [31] (pp. 145, 178–179).

In sum, by Dirac discontinuity, one cannot speak of a photon apart from an observed quantum phenomenon, even though, in the present view, a quantum object is still beyond any conception we can form and is only referred to as a photon on the basis of a certain determinate set of effects observed in measuring instruments. As indicated above, this claim or the concept of Dirac discontinuity would be more difficult to apply outright to the concept of a quantum field (rather than a particle considered as a wave) if a quantum field is considered as (physically) a quantum object, as it is in some, even most, especially realist, interpretations. This need not be the case, however. As I have discussed elsewhere, rather than as a quantum object, a quantum field can be defined, in accord with Heisenberg discontinuity, as an independent RWR-type reality responsible for quantum phenomena, and conversely, this reality can be defined as a quantum field, even, as noted above, in low-energy (QM) quantum regimes, but especially in high-energy (QFT) regimes [1,2]. In this view, only “particles” associated with a quantum field appear as quantum objects and, as such, are defined, by Dirac discontinuity, as idealizations applicable only at the time of observations. 

Is there a way, then, to rigorously define these two situations—described, as experimental arrangements, in terms of either only one or both channels open—as different? Yes, of course. The difference between them is defined by the different data registered in such events—as observed, as phenomena, in different, specifically complementary setups (which we can establish and control classically)—and by different probabilistic predictions, enabled by the formalism (cum Born’s rule) concerning these events. This difference, again, is often seen, as by Leggett, under the heading of the quantum measurement paradox. In the present view, the situation, rather than being paradoxical, obeys the logic of complementarity fully supported by the probabilities of quantum predictions. This is clearly suggested (without an appeal to Dirac discontinuity, at most only implicitly assumed) by Bohr’s definition of his concept of phenomenon:

I advocated the application of the word *phenomenon* exclusively to refer to the observations obtained under specified circumstances, including an account of the whole experimental arrangement. In such terminology, the observational problem is free of any special intricacy since, in actual experiments, all observations are expressed by unambiguous statements referring, for instance, to the registration of the point at which an electron arrives at a photographic plate. Moreover, speaking in such a way is just suited to emphasize that the appropriate physical interpretation of the symbolic quantum-mechanical formalism amounts only to predictions, of determinate or statistical character, pertaining to individual phenomena appearing under conditions defined by classical physical concepts [describing the observable parts of measuring instruments]. [5] (v. 2, p. 64).

Bohr, accordingly, saw the quantum-mechanical situation as indicating “the ambiguity in ascribing customary physical attributes to atomic [quantum] objects” themselves or to their independent behavior, as against phenomena, in his sense, something that is actually observed or registered. Instead, he concludes: “To my mind, there is no other alternative than to admit that, in this field of experience, we are dealing with individual phenomena and that our possibilities of handling the measuring instruments allow us only to make a choice between the different complementary phenomena we want to study” [5] (v. 2, p. 51).

It is crucial that effects defining phenomena must be considered under rigorously specifiable experimental conditions and that this specification must itself be seen as part of each phenomenon. Thus, if seen independently of one or the other complementarity context of its appearance, each mark on the screen in the double-slit experiment would be perceived in the same way or as the same phenomenon in the sense of philosophical phenomenology, from Kant to Husserl. Such a mark would appear the same regardless of the difference in the physical conditions and, hence, the statistical outcomes of the double-slit experiment. According to Bohr’s understanding, however, each mark is, or is part of, a different individual phenomenon depending on these conditions, the conditions of which are mutually exclusive in the case of complementary phenomena and are uniquely defined by each phenomenon in any circumstances. Thus, in the double-slit experiment, rather than dealing only with two phenomena, each defined by a different multiplicity of spots on the screen, we deal with two distinct multiplicities of individual phenomena, each defined by a spot on the screen. Each is an individual phenomenon in Bohr’s sense and depends on a different set of conditions of the experiment. One of these sets will lead to the emergence of the interference pattern, “built up by the accumulation of a large number of individual processes, each giving rise to a small spot on the photographic plate, and the distribution of these spots follows a simple law derivable from the [quantum-mechanical] wave analysis” ([5] (v. 2, pp. 45–46)). Wave analysis refers here to the use of the formalism in properly predicting the statistics of this outcome and not to any physical wave, to the mathematical “interference” found in the formalism, as each individual phenomenon is always discrete relative to any others. The other will not, and QM will correctly predict the probabilities in this case as well. Each spot must be seen as a different individual phenomenon defined by the conditions in which the event occurs, while two different patterns, “interference” and “no interference”, pertain to two sets of different individual phenomena. In either setting, there is always a nonzero probability for a given object to hit the screen outside the predicted range or structure (such as that of an interference pattern). Nevertheless, we should never mix considerations that belong to complementary experimental setups in analyzing a given experimental outcome, even when dealing with a single spot on the screen. 

The difference between two setups and their outcomes, individually or collectively, is defined by different experimental arrangements and different probabilities or statistics of the observed outcomes, properly predicted by QM. Nothing, however, refers to what actually happens to quantum objects between observations, which, moreover (and only them, in the present view), allow for the application of the concept of quantum objects or any specific quantum objects, such as electrons or photons. Each individual event only refers to a phenomenon, which, given the data registered, can be associated with one quantum object or another, which is still beyond conception itself. On the other hand, the setups define different probability distributions of the outcomes. The same type of considerations would apply to beam-splitter and other experiments, in which we deal with the “which-way” question, an expression no longer rigorously applicable. There is no unambiguous meaning to the “which way” question for the passing of a photon or any quantum object. However, there are unambiguously definable different probabilities and statistics for the registered outcomes, depending upon whether only one or both “ways” are open, part of the experimental setup to which, as described classically, we can apply such concepts.

What of interference, then, and what of the interference of a single photon with itself, of which Dirac spoke? Historically, the difficulty emerges with the introduction of the very idea of the photon by Einstein, as noted by Bohr:

Notwithstanding its fertility, the idea of the photon implied a quite unforeseen dilemma, since any simple corpuscular picture of radiation would obviously be irreconcilable with interference effects, which present so essential an aspect of radiative phenomena, and which can be described only in terms of a wave picture. The acuteness of the dilemma is stressed by the fact that the interference effects offer our only means of defining the concepts of frequency and wave-length entering into the very expressions for the energy [*E = h*ν, where ν is the frequency] and momentum [*P = h*σ, σ-wave length] of the photon [5] (v. 2, p. 34).

It thus became clear early on that either a “simple corpuscular picture” or a simple wave picture, and ultimately any form of either picture, could not apply even as complementary. This impossibility eventually, with QM and its mathematical concept of interference related to probability amplitudes, brought the situation to the stage to which Dirac refers by his statement: “Each photon… interferes only with itself”, he says, “Interference between different photons never occurs” [18] (p. 9).

Dirac is obviously correct on the second point, and he shows that assuming otherwise “would contradict the energy conservation” [18] (p. 9). That interference between different photons does not occur is also made clear by the fact that the interference pattern in the double-slit experiment would emerge even if the interval between each emission is sufficiently large for the next photon to be emitted only after the previous photon has hit the screen and has been destroyed by the collision. To assume the interference between different photons under these circumstances would amount, as noted by Leggett, to radical assumptions concerning the collective properties of photons as independent of their individual properties.

On the other hand, Dirac’s statement that “each photon… interferes only with itself” is not sufficiently explained by him. It may, however, be read as capturing something essential about the situation. The problem is, again, the language of “interference”, borrowed from classical wave physics. Dirac grounds his contention in the following point: “Some time before the discovery of quantum mechanics people realized that the connexion between light waves and photons must be of statistical character. What they did not clearly realize, however, was that the wave function gives information about the probability of *one* photon being in a particular place and not the probable number of photons in that place” [18] (p. 9). The statistical nature of the situation arises if we repeat the same experiments many times, that is, the experiment prepared with the same classical state of the observed parts of measuring instruments used, which is always possible by virtue of its classical nature. The statement concerning each photon interfering only with itself could then be read as follows, assuming, first, as Dirac did, that one can speak of a photon as a quantum object independently of an experiment, an assumption abandoned in this article by virtue of Dirac discontinuity but clearly made by Dirac. He does not appear to imply, especially given his overall analysis of the situation, that his statement is meant in a physical sense. Instead, it refers to the linear superposition of quantum states (as state vectors in the corresponding Hilbert space) defined by the wave function, which, again, deals with probabilities concerning the outcome of experiments [18] (pp. 11–14). The probabilities encoded in the wave function concerning our predictions concerning where each photon will hit the screen in the double-slit experiment correspond to the interference-pattern distribution of the traces left on the screen that will, inevitably, emerge in the corresponding setup. The probabilities are different in the alternative (no-interference-pattern) setup of the double-slit experiment, and they are, correspondingly, differently predicted by QM. On the one hand, speaking, as is done sometimes, of “probability wave interference” is at best misleading. A “probability wave” can only be used as a metaphor, accompanied by a proper explanation. Probabilities do not interfere or “propagate”, in the first place. We assign them, based on previous events of measurement, to discrete future events of measurement in accordance with (discrete) expectation-catalogs established by wave functions cum Born’s rule [17] (p. 154).

A clear example of the situation, as it appears in Bohr’s or the present interpretation, is the polarization of a photon (shifting, for the moment, to discrete variables, which does not change the essential point). There are two possible outcomes of measurement (after the initial preparation): for example, the horizontal state *H* and the vertical state V. In RWR-type interpretations, one could not say, as it is said sometimes, that before it is measured, the photon is (or is prepared) in a superposition of two physical states. The wave function allowing one to predict either physical state *H* or *V* is written as |ψ〉=α|h〉+β|v〉, with the probability amplitudes of |ψ〉 associated with state vector |h〉 given by α and |v〉 given by β. In a random experiment, the probability of the photon, when its polarization is to be measured, to be horizontally polarized is$\;{\left|\alpha \right|}^{2}$ and to be vertically polarized is ${\left|\beta \right|}^{2}$ (by Born’s rule). That, however, need not, and in the RWR view does not, mean that |ψ〉=α|h〉+β|v〉 represents the photon in a superposition of two physical states, *H* and *V***,** as nothing can be said concerning what happens between observations in the RWR view. Only the (mathematical) state vectors, designated |h〉 and |v〉 (in small letters) in the Hilbert space used, are in a linear (mathematical) superposition, with given amplitudes, and not quantum objects, in the present view, a concept only applicable at the time of measurement by Dirac discontinuity.

Dirac also says, however, that “[QM] gets over the difficulty [of assuming the interference between different photons] by making each photon go partly into each of the two components of equal intensity into which a beam of light is split [by a beam splitter device]” [18] (p. 9). As I argue here, this statement is difficult to sustain in this form, and Dirac does not appear to adequately support it. As stressed earlier, the beam splitters do not split individual photons; they split beams. On the other hand, as I argue here, it is equally difficult or even impossible to claim, logical as it might be in the absence of a photon splitting into two particles, that a photon goes through either one or the other channel when both are open to it. If it were a wave, a photon could be seen as going through both, but it is never detected doing so. In the present view, all of these statements are inapplicable or, as Bohr would have it, ambiguous, as is any statement concerning what “happens” between experiments. By Dirac discontinuity, the concept of a photon, an electron, or any quantum object, elementary or composite, is only applicable once the corresponding mark or trace is observed and, thus, the corresponding quantum phenomenon is established. As stated, however, one never observes more than one such trace in any individual run of the experiment. All detections happen after both channels are left behind, and they only detect particle-like events associated with individual photons, which, in RWR-type interpretations, does not allow one to speak of a photon as a particle either, any more than as anything else, even assuming that this concept only applies at the time of observation. On the other hand, the probabilities or statistics of what is observed are different depending on the setup. 

## 4. Conclusions

Building on Heisenberg’s and especially Bohr’s thinking, while also moving beyond it, this article offers a rethinking of the double-slit experiment and, by implication, other paradigmatic quantum experiments and of quantum phenomena and QM by means of, in terms of this article, the reality-without-realism (RWR) interpretation. This interpretation is defined by three main types of discontinuity—Heisenberg, Bohr, and Dirac discontinuities—which, I have argued, arise from and allow one to reconceptualize the essentially discontinuous nature of quantum phenomena. Reciprocally, however, this interpretation and these three forms of quantum discontinuity are justified by (along with other key quantum experiments) the double-slit experiment.

I briefly reprise each discontinuity. As their designations suggest, the concepts of Heisenberg and Bohr discontinuities were introduced by Heisenberg and Bohr. Heisenberg discontinuity, most essentially defining RWR-type interpretations, places the emergence of quantum phenomena at least beyond representation or knowledge, which is the weak form of such interpretations, or even beyond conception, which is the strong form of such interpretations, eventually assumed by Bohr’s interpretation of the one adopted here. “Bohr discontinuity”, introduced as part of Bohr’s interpretation under the assumption of Heisenberg discontinuity, grounds RWR-type interpretations, beginning with Bohr’s own, in the irreducible role of measuring instruments in the constitution of quantum phenomena while distinguishing these phenomena from quantum objects. Quantum objects, by Heisenberg’s discontinuity, were assumed by Bohr to be beyond representation and, ultimately, conception. By contrast, the behavior of the observable parts of measuring instruments, impacted by quantum objects and defining quantum phenomena, was idealized as representable, specifically by means of classical physics. Measuring instruments also have quantum strata through which they interact with quantum objects. 

On the other hand, the concept of “Dirac discontinuity”, which postulates that the concept of a quantum object, such as a photon or electron, is an idealization that only applies at the time of observation, is the main conceptual contribution of this article, in part following the earlier work by this author, cited earlier [1,2,3]. As I have argued here, adopting the concept of Dirac discontinuity is equally motivated by understanding, along the RWR line, low-energy quantum experiments, such as, in particular, the double-slit experiment. The reason for using the designation “Dirac discontinuity” is that, while not considered by Dirac himself, it may be seen as arising more naturally from his work on QED and especially his famous equation for the relativistic electron, arguably his greatest contribution to fundamental physics. Mathematically, Dirac’s equation made the wave function a four-component Hilbert-space vector, as opposed to a one-component or, if one considers spin, two-component Hilbert-space vector, as in QM, keeping in mind that each component is infinite-dimensional. These four components represent the fact that Dirac’s equation

$$\begin{array}{c} \left(\beta mc^{2}+\;\sum_{k=1}^{3}\alpha_{k}p_{k}c\right)\psi \left(x,t\right)=i\hbar \frac{\partial \psi \left(x,t\right)}{\partial t}\\ \alpha_{i}^{2}=\;\beta^{2}=I_{4} \end{array}$$

$$\begin{array}{c} \left(I_{4}\;\mathrm{is}\;\mathrm{the}\;\mathrm{identity}\;\mathrm{matrix}\right)\\ \alpha_{i}\beta +\beta \alpha_{i}=0\\ \alpha_{i}\alpha_{j}+\alpha_{j}\alpha_{i}=0 \end{array}$$

is an equation for both the (free) electron and the (free) positron, including their spins, which the equation contains automatically, in contrast to QM, where predicting the spin of an electron needs to be handled separately via Pauli matrices combined with Schrödinger’s equation. Dirac’s equation reflected and, as it happens, led to the discovery that a different particle (in the present view, again, defined in terms of effects observed in measuring instruments) can be registered in the course of a single experiment: the initial observation can register an electron, while the next one can register a positron, a photon, or an electron–positron pair, with the probabilities defined by the same equation. Once one moves to still higher energies, the panoply of possible outcomes becomes even greater. In the case of QED, we only have electrons, positrons, and photons; in QFT, depending on how high the energy is, one can literally find any known elementary particle or combination; that is, again, the corresponding effects will be registered. Heisenberg saw Dirac’s discovery of antimatter as “perhaps the biggest change of all the big changes in physics of our [twentieth] century. It was a discovery of utmost importance because it changed our whole picture of matter… It was one of the most spectacular consequences of Dirac’s discovery that the old concept of the elementary particle collapsed completely” [32] (p. 31). One of these changes, even if it was not considered by either Dirac, Heisenberg, or Bohr, was the concept of Dirac discontinuity, which, along with Heisenberg and Bohr discontinuities, defines the argument of this article and its rethinking, “yet once more”, of the double-slit experiment and, with it, the nature of quantum phenomena. I would not go so far as to claim that the RWR-type interpretation of quantum phenomena proposed here, as defined by Heisenberg, Bohr, and Dirac discontinuities, is the best explanation possible for the double-slit and other paradigmatic quantum experiments. I would argue, however, that these experiments provide strong support for this interpretation, an argument that was my aim in this article. 

I would like to close on a different note, which might seem unexpected given the preceding argument but which, in fact, shadowed it all along, beginning, by implication, with my title, “yet once more”, borrowed from John Milton. It is Milton’s context (one of his contexts) rather than the phrase itself, not so uncommon, that is significant here. It is remarkable and enigmatic and, as Heisenberg said, fortunate for us that the mathematics of QM and, in view of the more radical complexities ushered in by Dirac, QFT works so well. In the end, then, it is mathematics that, from Galileo to Heisenberg and beyond, moves theoretical physics forward in response to our interaction with nature by means of our experimental technology. It is mathematics that moves theoretical physics “to fresh woods and pastures new”, to cite the last words of Milton’s Lycidas (l. 194). “Yet once more”, borrowed by this article’s title, are the poem’s first words (l. 1). As Milton knew, however, and as Lycidas tells us, one must always make a decision about which direction to take—rarely a simple decision, as Milton knew as well. It was not for Milton or for Galileo, whom Milton visited in Florence and who figures in *Paradise Lost*, Milton’s greatest poem, or for Heisenberg. They did, however, find the right directions to move forward. Hopefully, we will too in confronting the problems that fundamental physics presents us with now, some of which, such as bringing together gravity, currently handled by general relativity, and other forces of nature, currently handled by QFT, are daunting. However, so were the problems that Galileo and Heisenberg confronted.

## Data Availability

Not applicable.
